# Exploring the Cost and Access Gap Between Osteopathic and Allopathic Medical Students When Applying for General Surgery Fourth-Year Away Rotations

**DOI:** 10.7759/cureus.80402

**Published:** 2025-03-11

**Authors:** Marc M Moza, Briley D Guarneri, Logan Morrison, LeAnn Allgood

**Affiliations:** 1 School of Osteopathic Medicine Arizona, A.T. Still University, Mesa, USA

**Keywords:** away rotation, away rotations, general surgery, medical student, osteopathic medical student

## Abstract

Introduction: Away rotations have been widely accepted as a critical factor involved in the selection of future residents by general surgery residency programs nationwide. With the recent change to a single accreditation system merging the American Osteopathic Association (AOA) and Accreditation Council for Graduate Medical Education (ACGME), programs offering away rotation positions must contend with an increased applicant pool consisting of both allopathic (MD) and osteopathic (DO) medical students. The aim of the study was to determine if any ACGME general surgery residency institutions discriminated against either osteopathic or allopathic students applying for away rotations.

Methods: All active, non-military ACGME-accredited general surgery residency programs (N=336) were reviewed. Official residency program websites, affiliated medical school websites, and the visiting student application service portal were all reviewed during the 2023-2024 application cycle. Eligibility criteria, associated fees, and specific verbiage were recorded and scored on a scale of zero to two. Additionally, we assessed for the acceptance of either the United States Medical Licensing Examination (USMLE) or Comprehensive Osteopathic Medical Licensing Examination (COMLEX), as mandated by allopathic and osteopathic schools, respectively. A score of two was given for unequal rotation fees between applicant degree types or language inhibiting students with a specific degree type from applying. A score of one was given to programs that only accepted USMLE scores and would not accept COMLEX scores or programs that only allowed students to apply from a limited list of osteopathic schools. A score of zero was given to programs that had no restrictive language.

Results: Out of the 336 programs reviewed, 52 did not have information regarding away rotations available on their respective websites, leaving 284 programs available for review. Of those 284 programs, 82.7% (N=235) were awarded a score of 0; 17.3% (N=47) received a score of either one or two. Among the subset of 47 programs, 40.4% (N=18) had a lexicon restricting students from Commission on Osteopathic College Accreditation (COCA) schools from rotating within the institution. 2.3% (N=1) had unequal monetary expenses between MD and DO applicants, with the latter group being charged 2500% more than the former; 59.6% (N=28) had specific language requiring USMLE scores while simultaneously stating their disapproval of COMLEX scores being used in their place. No programs (N=0) had discriminatory criteria (expense, board score, degree) directed toward MD applicants, and any disparities were only associated with DO applicants.

Conclusion: This study aimed to assess the degree of discrimination between MD and DO candidates applying for general surgery away rotations following the adoption of the single accreditation system. We highlight a subset of barriers that persist in the 'away rotation' application process that impacts allopathic and osteopathic applicants. Further elucidation of similar variables may be an essential target to recognize the value and contributions of both DO and MD applicants to general surgery residency programs.

## Introduction

In 2020, the American Osteopathic Association (AOA) and Accreditation Council for Graduate Medical Education (ACGME) completed a merger into a single accreditation system (SAS). The merger aimed to enhance choices and opportunities for residents, promote collaboration among the medical education community, and ensure consistency in evaluation methods and accountability standards [1]. Paradoxically, osteopathic (DO) students have since faced a steeper disadvantage at matching into more competitive specialties, such as general surgery, than their allopathic (MD) counterparts [2].

General surgery is considered one of the more competitive specialties to successfully match. In the 2023 National Residency Matching Program’s (NRMP) match, 5,132 applicants contended for only 2,803 available postgraduate year-one (PGY-1) general surgery positions, exemplifying such competition. This was followed by a match rate of 87.8% for MD seniors and 56.3% for DO seniors. Of those MD and DO seniors who ranked general surgery as their only choice, 11.9% and 33.6% went unmatched, respectively [3]. The surplus of applicants per available position led to 57.7% of all applications being rejected in standardized screening in 2022. Furthermore, 48% of programs admitted to seldom or never interviewing DO students, while only 3% did so for MD candidates, indicating a significant disparity in interview practices [4]. This data suggests that DO applicants are predisposed to be less likely to match into general surgery given fewer interview opportunities.

Achieving a successful match into general surgery is often associated with strong United States Medical Licensing Examination (USMLE) Step Two Clinical Knowledge (CK) scores, research experiences, and away rotations [5]. Additionally, the recent transition of USMLE Step One to a pass-fail format creates an additional barrier for DO students to distinguish themselves; therefore, applicants must look elsewhere to do so. Away rotations, otherwise known as audition rotations, sub-internships, and externships, have been widely accepted as a key component of a general surgery applicant’s portfolio. Participation in an away rotation is associated with increased odds of matching by a factor of 1.68, as well as higher rates of interviews [6]. The importance of away rotations may be more significant now than ever before as a result of the ACGME merger. They may be especially essential to the DO student since 75% of osteopathic medical schools are not affiliated with a general surgery residency program [7].

The transition to a single accreditation system has shown that integrating DOs into ACGME surgical residencies will be a gradual process. As the SAS's success in standardizing surgical training seems to carry negative implications for osteopathic applicants, the role of the away rotation may prove even more valuable than in previous years [8]. Therefore, the aim of this study was to determine if any ACGME general surgery residency institutions discriminated against either osteopathic or allopathic students applying for away rotations.

## Materials and methods

Eligibility criteria

We included all active, non-military ACGME-accredited general surgery residency programs in the United States for the 2023-2024 application cycle. The inclusion criteria consisted of programs offering away rotations and having information available either through their official websites, the Visiting Student Learning Opportunities (VSLO) platform, or the Clinician Nexus portal. Programs were excluded if they did not provide accessible information about visiting student policies or away rotations on either the website or the application platforms (VSLO/Clinician Nexus). The study focused on determining bias or discriminatory criteria based on the applicant's degree type (MD vs. DO) and the requirement of specific licensing exams (USMLE vs. COMLEX).

Information sources

The primary data sources included the official websites of ACGME-accredited general surgery residency programs, the VSLO platform, and the Clinician Nexus portal. Data collection occurred between May 2023 and September 2023.

Search strategy

The search involved visiting each program’s official residency website, focusing specifically on sections relevant to visiting students, medical students, or Graduate Medical Education (GME). Where necessary, VSLO and Clinician Nexus portals were also searched for program eligibility criteria under the "elective available to" sections. We recorded information on whether the program accepted MD and DO applicants equally, required USMLE scores, or imposed differential application fees.

Study selection

A total of 336 programs were initially identified and assessed for eligibility. Programs were excluded from the analysis if no information regarding away rotations was available on either the program website, VSLO, or Clinician Nexus. This left 284 programs for further evaluation. The screening and eligibility assessments were performed independently by multiple authors to ensure consistency and resolve discrepancies through discussion.

Data collection process

Two independent reviewers extracted the data from the identified sources (websites, VSLO, Clinician Nexus) using a predefined data extraction form. Discrepancies between reviewers were resolved through discussion and consensus. We collected information on eligibility criteria, specific verbiage related to degree-type preference (MD vs. DO), examination requirements (USMLE vs. COMLEX), and fees associated with applying for away rotations.

Data items

The data extracted included degree type acceptance (MD, DO, or both), licensing exam requirements (USMLE, COMLEX, or both), fee structures for away rotations and whether fees differed based on degree type, as well as any restrictive language precluding applicants from Commission on Osteopathic College Accreditation (COCA) schools.

Summary measures

Each program was assigned a score based on the presence or absence of discriminatory criteria. A score of 0 indicated no discriminatory language or criteria were identified. A score of 1 was assigned to programs that required USMLE scores but did not accept COMLEX scores or that limited eligibility to osteopathic schools. A score of 2 was given to programs with explicit bias, such as unequal fees for MD versus DO applicants or restricted access for non-Liaison Committee on Medical Education (LCME) students.

Synthesis of results

A quantitative synthesis was conducted by summing the scores across programs and calculating the percentage of programs with discriminatory practices. Results were stratified based on geographic region (West, Southwest, Southeast, Midwest, and Northeast) to examine regional disparities in program criteria.

Regional analysis

Programs were categorized into five geographic regions (West, Southwest, Southeast, Midwest, and Northeast) to explore potential regional disparities in discriminatory practices against DO applicants. The data were analyzed to identify any trends or patterns in specific regions.

## Results

Description of residency programs

A total of 336 ACGME-accredited general surgery residency programs were initially reviewed. Among these, 52 programs did not furnish information regarding away rotations on their respective websites. This omission left 284 programs available for comprehensive analysis.

Scoring distribution

Upon meticulous evaluation, it was observed that the majority, 82.7% (n=235), of the programs received a score of 0, denoting an absence of discriminatory criteria or restrictive language in their away rotation application processes. However, 17.3% (n=47) of the programs received a score of one or two, indicating some level of disparity or discrimination against either MD or DO applicants.

Discriminatory criteria

Among the subset of 47 programs exhibiting discriminatory criteria, 40.4% (n=18) had lexicon restrictions, thereby hindering students from non-Liaison Committee on Medical Education (LCME) schools from participating in rotations within the institution. A minute percentage, 2.3% (n=1), was found to impose unequal financial burdens, with DO applicants charged at a rate 28 times higher than their MD counterparts. Furthermore, 59.6% (n=28) of these programs required USMLE scores while not recognizing COMLEX scores, despite COMLEX being a licensing requirement for DO students just as USMLE is for MD students. 

Detailed discriminatory practices

Lexicon Restrictions

Programs implementing lexicon restrictions employed language that precluded students from non-LCME schools, thereby restricting the pool of potential applicants based on their medical school's accreditation.

Financial Disparities

A solitary program (2.3%) was discovered to impose substantial monetary barriers, notably favoring MD applicants by levying fees 28 times higher for DO applicants.

USMLE vs. COMLEX Requirements

Nearly 60% of the programs with discriminatory criteria explicitly stipulated the necessity of USMLE scores, potentially disadvantaging DO applicants who may possess COMLEX scores instead.

Regional bias analysis

**Figure 1:**
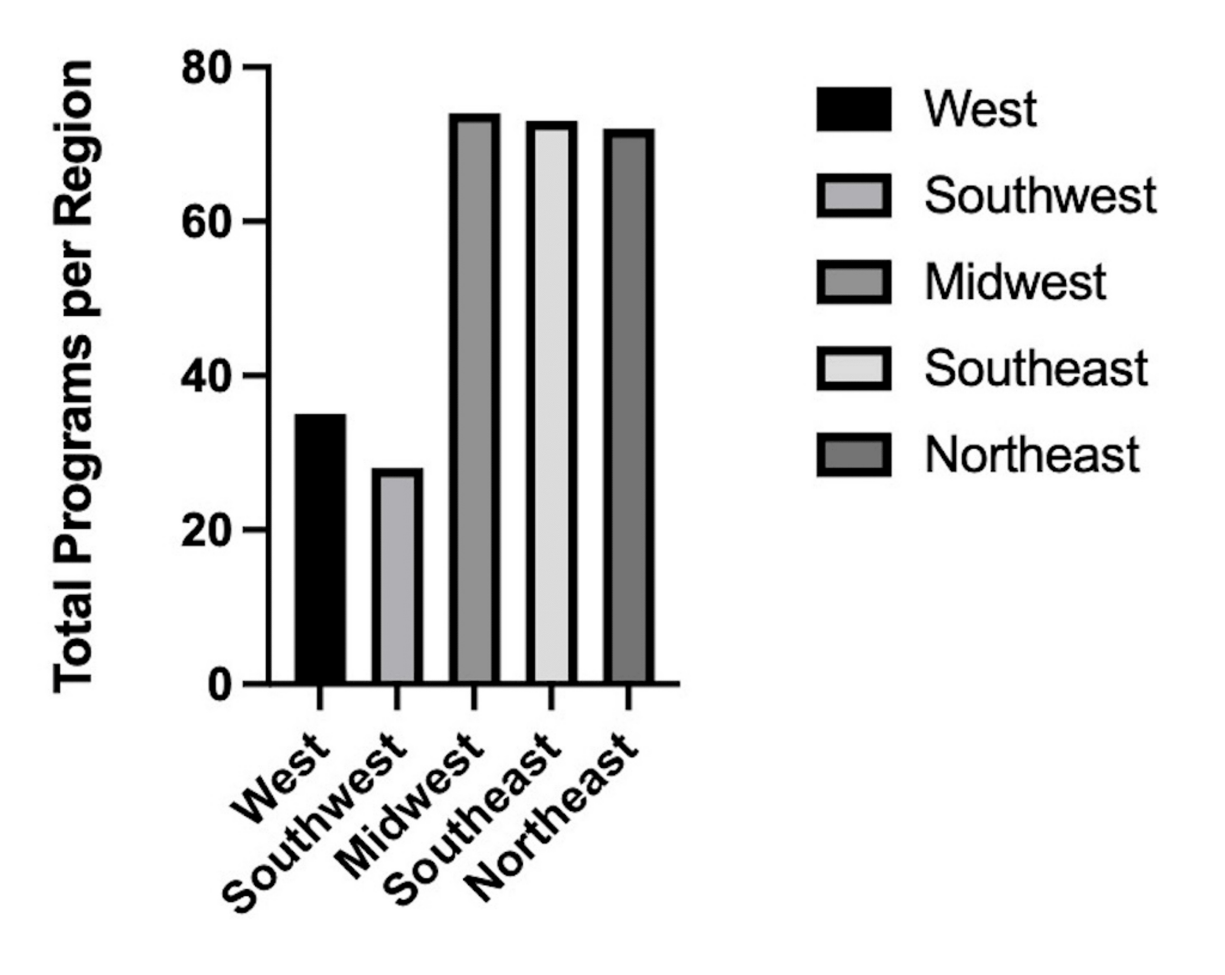
Total programs allocated to each region.

**Figure 2:**
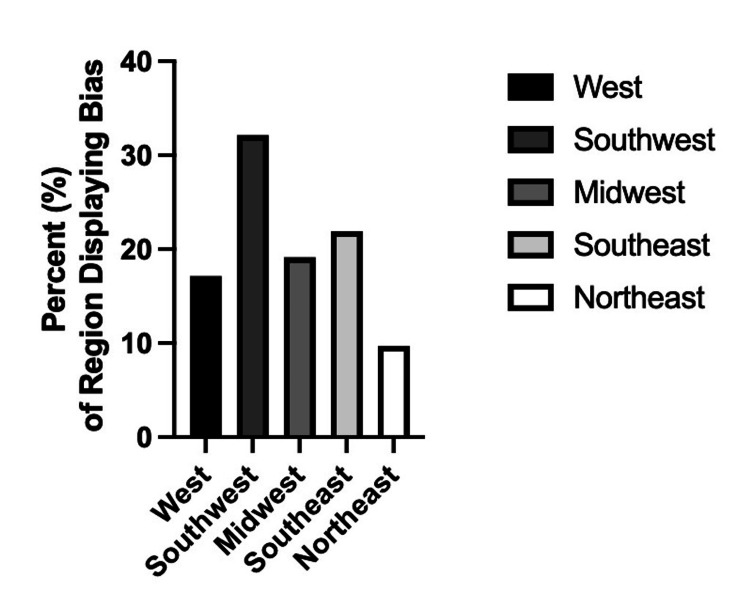
Percentage of general surgery residency programs by region which display any form of bias towards DO candidates when applying for fourth year away rotations. DO: Osteopathic

An examination of regional disparities in the away rotation criteria reveals notable variations across different geographic regions (Figures 1, 2). Among the total programs reviewed, the highest raw total of discriminatory criteria was observed in the Southeast region, where 16 out of 73 (21.9%) programs exhibited bias towards either MD or DO applicants. Similarly, the Southwest region demonstrated a considerable prevalence of discriminatory criteria, with 9 out of 28 (32.1%) programs imposing restrictions. In the Midwest and Northeast regions, 9 out of 74 (12.2%) and 7 out of 72 (9.7%) programs, respectively, displayed biases in their away rotation application processes. The West region exhibited relatively lower levels of bias, with 6 out of 35 (17.1%) programs showing discriminatory criteria. These findings suggest that regional disparities exist in the treatment of MD and DO applicants, emphasizing the need for further investigation into the underlying factors contributing to regional variations in away rotation policies.

Programs with no discriminatory criteria

The overwhelming majority, constituting 82.7% of programs, exhibited no discriminatory criteria or restrictive language. These programs provided an equitable platform for both MD and DO applicants, fostering a level playing field in the residency application process.

## Discussion

The merger of the ACGME and AOA became a milestone in the mission to standardize medical training and enhance resident education opportunities. This consequentially crowded out prospective DO residents from more competitive specialties, such as general surgery [9]. It is unclear whether these disparities existed before the merger or were created after the fact. What is evident, however, is that it is difficult or impossible for DO students to obtain an away rotation at 17.3% of general surgery residency institutions. This raises questions as to whether this disparate access is intentional. If so, is it reasonable for institutions to overlook COCA-accredited students by only accepting students from schools following LCME accreditation guidelines? We pose that any implicit bias may have been illuminated by the merger once these former MD-only programs were required to add DO students to their pool of applicants. Subsequently, the ramifications of the formation of a single accreditation body have been detrimental to DO applicants thus far and will continue to be evaluated by future match data [10].

Notably, in the years since the merger, we have seen that the DO applicant remains at a consistent disadvantage in matching into general surgery in comparison to their MD counterparts [8]. This merger has introduced additional challenges to the osteopathic community, including potential biases and reduced recognition of osteopathic training within residency selection processes [11]. Alongside the new pass/fail format of USMLE Step 1, this has created a heavier emphasis on other distinctive portions of an application, including away rotations. The away rotation has proven to be a valuable component of any medical student’s application to general surgery [6]. The low rates at which DO students are matching into general surgery may in part be due to a fewer number of programs offering away rotations to these students. Thus, this study aimed to assess whether ACGME general surgery residency institutions discriminated against either osteopathic or allopathic students applying for away rotations. 

Remarkably, one program imposed a fee of $4,150 for osteopathic students and $150 for allopathic students, likely to deter the former from applying [12]. This specific program cites their reasoning as the lack of reciprocality for MD students to rotate at osteopathic institutions, though there is no known evidence of these claims. Additionally, our findings suggest that regional disparities may exist in the treatment of MD and DO applicants, emphasizing the need for further investigation into the underlying factors contributing to regional variations in away rotation policies. Future research should be targeted toward any variability in away rotation offerings and how these may impact the match rate discrepancies into general surgery residency programs among MD and DO students. The identification of the prevalence of similar discrepancies in other specialties should also be investigated.

## Conclusions

Despite the fact that a majority of programs provided equal opportunities to students of both degrees, there was still a significant portion that discriminated solely against DO students. As both MDs and DOs are able to obtain full licensure throughout the US while they work alongside each other in all medical specialties, it is important that all medical students share the same opportunity to pursue away rotations. These away rotations are potentially beneficial for both the program and the student, as they provide an opportunity to gauge the 'fit' as a potential resident. With the still relatively recent move to an SAS, it is important for the ACGME and residency programs to consider equitable opportunities for all students. Raising awareness of these disparities is a crucial step in ensuring that accreditation bodies recognize the issue and subsequently identify strategies to ensure equal access for all students. 
